# When Maintaining Relationships and Social Connectivity Matter: The Case of New Zealand Midwives and COVID-19

**DOI:** 10.3389/fsoc.2021.614017

**Published:** 2021-03-16

**Authors:** Susan Crowther, Robyn Maude, Billie Bradford, Diana Austin, Andrea Gilkison, Judith McAra-Couper, Jayne Krisjanous

**Affiliations:** ^1^ Centre for Midwifery and Women’s Health Research, Faculty of Health and Environmental Studies, AUT University, Auckland, New Zealand; ^2^ School of Nursing, Midwifery, and Health Practice, Te Herenga Waka Victoria University of Wellington, Wellington, New Zealand; ^3^ Wellington School of Business and Government, Te Herenga Waka Victoria University of Wellington, Wellington, New Zealand

**Keywords:** midwives, New Zealand, COVID-19, continuity of care, lockdown, choice, media analysis, community

## Abstract

New Zealand’s response to COVID-19 was go hard and go early into Level 4 lockdown on 25^th^ March 2020. This rapid response has resulted in low rates of infection and deaths. For New Zealand midwives, the sudden changes to how they work with women and families during pregnancy, birth and postnatally, especially in the community, required unprecedented innovation and adaptation. The volume of information coming from many different sources, and the speed with which it was changing and updating, added further stress to the delivery of a midwifery model of care underpinned by partnership, collaboration, informed choice, safety and relational continuity. Despite the uncertainties, midwives continued their care for women and their families across all settings. In the rapidly changing landscape of the pandemic, news media provided a real time account of midwives’ and families’ challenges and experiences. This article provides background and discussion of these events and reports on a content analysis of media reporting the impact on the maternity system in New Zealand during the initial surge of the COVID-19 pandemic. We found that the New Zealand midwife was a major influencer and initiator for relational care to occur uninterrupted at the frontline throughout the COVID-19 lockdown, despite the personal risk. The initial 5-week lockdown in March 2020 involved stringent restrictions requiring all New Zealanders, other than essential workers such as midwives, to remain at home. Midwives kept women, their families and communities central to the conversation throughout lockdown whilst juggling their concerns about keeping themselves and their own families safe. Insights gained from the media analysis suggest that despite the significant stress and upheaval experienced by midwives and wāhine/women, relational continuity facilitates quality and consistent care that honors women’s choices and cultural needs even during situations of national crisis.

## Introduction: The New Zealand Response

The New Zealand (NZ) response to the initial surge of the COVID-19 pandemic was swift and decisive, resulting in a hard early lockdown, before any deaths occurred. This approach meant that NZ achieved low rates of infections and deaths compared to other high-income countries. These low rates of infections and mortality are due to the adoption of a set of early non-pharmaceutical interventions that explicitly focused on bringing COVID-19 incidence to zero (Jefferies et al., 2020). Transmission rates were kept low by a governmental coordinated national response that interrupted transmission chains centrally and locally across the country. The highest incidences were in popular tourist areas and at large events such as weddings; these were named “clusters.” The stringent early lockdowns stopped large gatherings, travel restrictions were imposed, and the geographical location of NZ enabled borders to be closed. These measures helped contain the emergence of new clusters. At the time of writing (January 2021), anyone entering NZ is required to remain 2 weeks in a managed isolation quarantine (MIQ) facility and have two negative COVID-19 tests before entering the general population. New Zealand’s success in the pandemic to date stems from early decisive government-led responses including robust surveillance systems, accessible testing and quarantine processes (Robert, 2020).

However, the suddenness and extent of the response had implications for the New Zealand midwifery workforce, whose overarching principles of care are partnership, collaboration, safety and relational continuity (Pairman and McAra-Couper, 2015). These principles are at the heart of *Te Tiriti o Waitangi*, New Zealand’s founding document, of *Te Ao Māori* (the Māori world) and of New Zealand midwives’ commitment to cultural safety (Farry and Crowther, 2014). Choice, safety and maintaining social connectivity are core values that midwives practicing in NZ sought to ensure for all women throughout the COVID-19 response. COVID-19 represented a defining moment for the NZ midwifery workforce, placing midwives in a quickly evolving situation requiring rapid accommodation of changes to practice.

The New Zealand government developed a 4-level Alert System to communicate restrictions in relation to the level of COVID-19 risk. The most stringent restrictions were at Alert Level 4, which required all New Zealanders, other than essential workers, to remain at home. During an almost 5-week lockdown starting on 25^th^ March 2020 (MoH, 2020a), midwives continued to provide care to women and their families across all settings. Births at home and in standalone birth centers continued, and, in some cases demand reportedly increased. Yet much of this midwifery work continued unseen, within a system where midwifery consistently finds itself under-resourced, underpaid and relatively invisible to the government (NZIER, 2020).

The situation created by the COVID-19 pandemic highlighted the relative invisibility of midwives as frontline essential workers, despite continuous efforts by the NZ College of Midwives (NZCOM) to advocate for midwives. The constantly changing guidance from myriad sources from governmental, regulatory and district health boards, and difficulties accessing personal protective equipment (PPE) for all primary care providers, especially midwives—50% of whom practice in the community—in homes, standalone birth centers, and small community hospitals—imposed further stress on an already personally stressful pandemic reality. This article discusses evidence on midwives’ and childbearers’ experiences, with focus on an analysis of media reports on midwives and maternity in New Zealand during the initial surge of the COVID-19 pandemic. By analyzing how the issues, challenges and experiences, as narrated by relevant voices (midwives, maternity healthcare co-ordinators, wāhine/women and their whānau/families (these are Maori terms, now commonly used across New Zealand) in a rapidly evolving situation, we further illuminate the complexity and efforts to maintain the roles and relationships with women that New Zealand midwives upheld. In particular, our analysis reveals the near-invisible work midwives do in the community and how this work is frequently undervalued by policy makers. O’Connell et al. (2020) sent out an international call for midwifery solidarity during the emerging pandemic. This show of solidarity was clearly evident in the New Zealand context through the initiatives midwives took to mitigate the consequences of lockdown, including the leadership shown to support and make sure all childbearers and midwives were safe (NZCOM, 2020b).

## The New Zealand Maternity Context

New Zealand has a population of 4,951,500, with 59,637 births per year (Stats NZ, 2020). At the end of 2019, 3226 midwives held practicing certificates in NZ (Midwfery Council, 2019). Midwives in NZ are educated over a 4-year degree program at five tertiary institutions (Gilkison et al., 2016). Once registered, a NZ midwife is required to renew her practicing certificate annually. Midwives practice either as “core” midwives working as employees in hospitals or standalone birth centers or as “caseload” midwives—self-employed community-based midwives providing continuity of care from early pregnancy to 6 weeks postpartum within an established and integrated maternity care system. Caseload midwives predominantly work in the community and are called Lead Maternity Carers (LMCs). Most LMCs are midwives, although they can also be family physicians and obstetricians; currently 94.2% of women choose a midwife as their LMC (MoH, 2019). Core and caseload/community midwives attend births together in hospitals and birth centers; they also often shift between these two roles. For example, a community midwife with young children may choose to work as a core midwife on shift for the regular hours, then change back to caseload work when those children are older. This frequent role-switching helps to ensure that all midwives maintain a shared philosophy of care. All maternity care in NZ, including midwifery LMC care, is fully government-funded and free to NZ citizens and residents, unless the childbearer chooses a specialist obstetrician (Guilliland and Pairman, 2010).

The overarching premise is that women choose and know the midwife who will work with them throughout their childbearing experience. Women access midwives through word of mouth, the internet, and referral from hospitals and family physicians. LMC midwives have caseloads ranging from one to eight women each month depending on region (e.g., variation in urban/rural/remote rural). An LMC midwife caring for four to six women per month is understood as practicing full time and needs to ensure 24/7 on call by herself or an arranged backup. LMC midwives are encouraged to work in group practices to moderate the constant 24/7 on-call commitment. New Zealand maternity care is women-centered, acknowledging pregnancy and birth as normal life events (MoH, 2011). The *midwifery philosophy of partnership* is based around communication, negotiation, equality, shared responsibility and empowerment, and informed choice and consent (Guilliland and Pairman, 2010). This midwife-wāhine/whānau collaboration acknowledges a sharing of each other’s knowledge, experiences, skills, and feelings. Relationships with women and their families facilitate informed decision making and are identified as an attribute of autonomous midwifery practice that motivates New Zealand midwives to advocate for women across all practice settings and circumstances (Clemons et al., 2020). Midwives’ expertise in building and sustaining relationships is intrinsic to this autonomy. Relational continuity is demonstrated through communication and negotiation, thereby building a trusting relationship over time with the client, her whānau and the community; together these elements provide the safety and acceptability of midwifery services (Davies and Crowther, 2020).

New Zealand maternity services are held in great regard internationally due to their high rates of maternal satisfaction and breastfeeding initiation and lower rates of cesarean births as compared to other high income countries (Rowland et al., 2012). Infant death has decreased by 41% from 7.3 to 4.7 between 1996 and 2017 (MoH, 2020b) and there has been an impressive reduction in stillbirths between 2007 and 2015 (PMMRC, 2018). The NZ stillbirth rate of 2.3 per 1,000 was reported in the *Lancet Stillbirth Series* at 10^th^ lowest in the world (Flenady et al., 2016).

There are, however, ongoing concerns about the economic sustainability of the NZ midwifery model of care. A recent report prepared by the NZ Institute of Economic Research (NZIER) for the NZCOM highlighted pay equity as an “underlying factor in the sustainability of improving perinatal health outcomes” (NZIER, 2020, i). In addition, problems with midwife retention and staff shortages in some sectors and regions further exacerbate issues with workforce sustainability (NZIER, 2020). However, these reports of staff shortages are at odds with figures from Midwifery Council of NZ that reveal high numbers of midwives with current annual practicing certificates and increasing numbers of graduates in 2018/2019 (MCNZ, 2019). This discrepancy might be related to the ongoing struggles of midwives for pay equity when compared with others with comparable accountability and responsibility in their professional roles, such as obstetricians and family doctors, resulting in an increasingly vocal call to value the work of midwives (Berinstein et al., 2020).

To appreciate the resentment that such discrepancy causes, it is important to note the comprehensive and autonomous role of the midwife in New Zealand maternity services. The New Zealand midwife is responsible and accountable to the public and for the care she provides to the women in her caseload, including prescribing and administering medications within her scope of practice; to order, interpret and make decisions on many diagnostic and screening tests; perform full examination of the normal neonate; and repair most cases of perineal trauma. Although midwives’ maternity care may occur in conjunction with consultation and/or transfer of aspects of care to medical colleagues when complex biomedical concerns arise, for the most part, the midwife continues to coordinate care throughout a woman’s maternity experience. Another point of difference compared to other regions is that New Zealand midwives cannot be sued by women in their care, although a midwife can be held to account by the Midwifery Council of New Zealand for providing a poor standard of care (MCNZ, n.d.).

A long-anticipated announcement of increased funding for maternity services, particularly primary services in the 2020 national budget did not occur, to the great disappointment of the profession. However, in June 2020, during COVID-19 lockdown, community LMC midwives were compensated with a one-time payment of NZ$2500 for extra expenses incurred during the pandemic (Herald, 2020). Better remuneration of midwives continues to be an ongoing political issue that has now moved beyond just pay equity for midwives to also highlight the need to strengthen primary and community midwifery services. With midwives providing both hospital- and community-based care to women with increasing social and medical complexities, there is recognition that investment into primary maternity services, including midwifery, will have positive long-term benefits for maternal and child wellbeing (HDSR, 2020).

### Lockdown and NZ Midwifery

On March 25th, 2020 the NZ government announced a state of emergency and the country moved to Alert Level 4 full lockdown.^1^ The local and national implications were an immediate move to:

1.Everyone staying at home in their “bubble.”
2.Everyone to maintain 2 m apart when out of the home.
3.Only essential personal movement (e.g., health concerns, groceries).
4.Only safe recreational activity allowed in a local area.
5.All travel severely limited between regions and country borders closed.
6.All gatherings cancelled and all public venues closed.
7.Businesses closed except for essential services (e.g., supermarkets, pharmacies, clinics, petrol stations) and lifeline utilities.
8.All educational facilities closed.
9.Rationing of supplies and requisitioning of facilities as possible.
10.Reprioritization of healthcare services.

This was a rapid, decisive response, meaning the nation had just 48 h to organize all contingencies, including the logistics of maternity care provision. With much of the government and health system focused on preparing and equipping hospitals across the country for a potential torrent of COVID-19 admissions, frontline workers such as community-based midwives struggled to be seen and heard. Yet almost 5,000 babies were born during the 4 week period at Alert Level 4.

The early stages of the lockdown created the greatest degree of overwhelm, which was highlighted through NZCOM online forums and several social media chat sites. These forums foregrounded midwives’ feelings of being ignored and having their concerns not taken seriously, particularly around what midwives experienced as poorly managed policy and guidance directives and a paucity of necessary equipment such as PPE. Their unease with the unfolding countrywide lockdown was compounded by the constantly changing guidance from myriad sources, both local and international, and difficulties of access to PPE in an already stressful situation.

A significant point of difference in the New Zealand context is that a large part of funded NZ midwifery care is provided in the community, including antenatal and postnatal care, as well as primary birthing (e.g., birth at home and in standalone birth centers). The COVID-19 lockdown brought a host of very real challenges for the NZ midwifery workforce as they continued to provide continuity of care in the community, despite potential risks to themselves, colleagues and families in the context of fear about the extent of community transmission. As most New Zealanders stayed at home in their “bubbles,” community-based midwifery care continued across all settings, and indeed workload increased as women avoided going to hospital unless absolutely necessary. The increased community focus was caused by more home-based prenatal care and the increased demand for community births and for early discharge from hospitals for community postnatal care. (It must be noted that exact rates of this increased community activity are still being gathered at the time of writing). Choice, safety and maintaining social connectivity were core values that midwives sought to ensure for all women in all settings. In the rapidly changing landscape of the pandemic, news media provided real-time accounts of midwives’ and families’ challenges and experiences.

It became evident that there was much to learn about how to better prepare for any future pandemic and how to highlight the uniqueness of the NZ model of midwifery care, which consistently prioritizes and attunes to relationships and nurturing social connectivity for women, whānau, communities and their midwives. Although this spirit of generosity has been shown to help sustain NZ midwifery practice (Hunter et al., 2016), paradoxically, in the early part of the COVID-19 lockdown, this generosity of spirit appeared to be exploited. Initially, support came from within the profession itself. What was problematic and frustrating was that the organizational structures of the healthcare system appeared not to be listening and simply did not appreciate that babies would continue to be born during a pandemic, with inevitable additional workload demands on midwives. Yet over time, media outlets and social media platforms highlighted the work and contributions of midwives and their situation became progressively recognized. Consequently, we decided as a collaboration of NZ midwifery researchers to capture and report their voices for this Special Issue on *The Global Impacts of COVID-19 on Maternity Care Practices and Childbearing Experiences.*

## Methods: Media Content Analysis

To capture the relevant voices and discourses during the pandemic and to understand how the media reported the impact of COVID-19 on midwives and maternity care, we conducted a qualitative content analysis of NZ media articles between December 2019 and July 25, 2020. Content analysis enables meaningful insights and interpretations to be drawn from textual data and is a useful tool to explore the role of midwifery in the context of a pandemic, as it provides the opportunity to systematically analyze a broad platform of commentary and reporting of news and events as they are occurring. While online news websites and journalism are popular press media and therefore may not be as reliable as, for example, actual ethnographic research, the benefits of wide audience reach offer a compelling reason for analysis of information content disseminated during an acute event such as COVID-19. We prioritized news websites over social media, as while social media is useful to analyse sentiment, is not a medium often used as an entry-point to a news item (Vermeer et al., 2020).

### New Zealand Media and Search Strategy

Within NZ, print and online news outlets are governed by a duopoly between New Zealand Media and Entertainment (NZME) (publicly owned with main brands *NZ Herald* and Newstalk ZB) and Stuff (a publicly listed company). Stuff and the *NZ Herald* dominate audience share in online news (Myllylahti and Baker, 2019). We used the Stuff (stuff.co.nz) website—NZ’s largest media website—to search for relevant material. Stuff owns nine major NZ newspapers, including the *DomPost* (Wellington circulation) and *The Press* (Canterbury circulation). In electing to use the Stuff website as our data collection site, we did omit a major NZ newspaper, *The Herald*, which has an Auckland circulation as well as further online readership throughout NZ. However, given the coverage of all New Zealand regions on the Stuff website, we determined that use of this website provided a robust way of representatively capturing news that New Zealanders would have been exposed to during the search period.

Using online media sources is also appropriate given that print media (newspaper, journal and magazine) circulation and subscription are decreasing, with most newspapers also offering an online source to complement print (Myllylahti and Baker, 2019). A sweep of *The Herald* to ensure that we were not missing key items not being reported on Stuff gave us confidence that conducting analysis of the Stuff website only would give us robust qualitative insights. The comparative count and weighting of topics discussed on the *Herald* website vis a vis the Stuff website was a reassuring equivalent. We note however that we do include reference to NZ *Herald* articles (along with quotations) that we deemed useful in describing midwives’ reactions to a lack of PPE and increased workload.

Additionally, we conducted a search for articles printed in popular press magazines, particularly women’s lifestyle magazines (*Woman’s Day NZ* and *New Zealand Women’s Weekly*) using Pressreader, Te Waharoa and Ebsco databases. No print articles were retrieved for our search period. This may have been due to Bauer Media NZ closing their New Zealand business during the COVID-19 lockdown, disrupting production and publishing of these and several other New Zealand print magazines. Initial treatment of retrieved articles involved reading each online article to ensure that it was valid for our research. Of the 89 articles that we reviewed, five were rejected as they featured stories from overseas (mostly about transmission of COVID-19 to newborns in other countries). A further 12 were removed from analysis as they were considered irrelevant to the research question about COVID-19 and its effect on NZ midwives. One article was a repeat. Ultimately, a total of 71 pertinent articles were retrieved^2^ for analysis.

### The Analysis Process

To undertake the content analysis, we downloaded all article links to an Excel spreadsheet. Coding was undertaken by co-authors Jayne Krisjanous and Diana Austin, who also provided their spreadsheet for scrutiny and feedback to other authors. These same co-authors conducted the analysis. Both consulted each other on what they had found, and impressions gained. Final themes were derived through a consensus between these two co-authors, who again presented their findings for feedback and any reiteration to the other authors. In order to analyze the 71 articles, a systematic process was applied that incorporated first identifying major categories (first order codes), followed by a second stage that identified themes (second order codes) within each of the major categories. When identifying the general themes within the article, rather than prime or drive themes by key words, we chose any relevant discourse as it was presented to its audience. Where several themes were present within one article, we allocated these accordingly.

## Findings: Wāhine, Whānau, and Midwives

**FIGURE 1:**
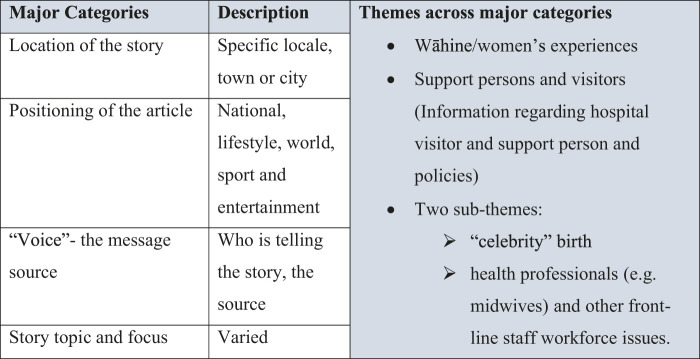
Themes from media analysis.

The major categories identified were: 1) region or location where the events in the story had occurred; 2) categories within the Stuff website to which the article was allocated (e.g., national news, lifestyle, entertainment or sports); 3) key message source of information or opinion; and 4) story topic and focus. A second stage involved breaking the major categories into themes (second order codes). Four main themes emerged as most salient: the client (women’s and their whānau (family) experiences; information regarding hospital visitor and support persons and policies; and two smaller categories, one on celebrity birth and another related to health professionals and other front-line staff workforce issues (*see* Figure 1). Here we report on the client, visitor/support persons and midwife findings.

### Wāhine/Women’s Experiences

For the wāhine/women’s theme, the quoted message source was almost always the woman/client. Sometimes partners (in this case, almost always the baby’s father) also “spoke,” particularly about their experiences. For articles about midwives, the NZCOM was consistently quoted (apart from two articles where an individual midwife was the key source). Another body of reporting tracked the movement of changes in hospital policies and visiting hours. During the period at Alert Level 4, regulations were communicated in a stringent tone; for women this meant that just one support person could be present for the birth and was required to leave soon after. (As other articles in this collection demonstrate, New Zealand appears to be unique in never completely prohibiting the presence of at least one support person.) As the country moved to lower Alert Levels and restrictions eased, the readership was informed of changes (e.g., from a single support person at the birth only to the addition of one visitor “per day” whilst an inpatient). For articles about hospital polices, a hospital spokesperson was the message source.

The client experience theme further broke down into women’s emotional responses to the upheaval, location of birth (home, small rural maternity hospital, standalone birth center, or large hospital), and the impact on fathers. Most media attention was given to women’s experiences overall. Women described birth during the Alert Level 4 period as “scary,” “negatively affecting mental health,” and “fraught” when in hospital, due to their partner having to leave soon after the birth. Although client experience articles rarely mentioned the midwife—a community or core (hospital or birth center) midwife—it is valid to assume that a midwife would have been influential in some way to overall impressions formed through her need to provide support in the absence of others, reconfigure service delivery and cope in potentially stressful environments.

Women indeed felt the absence of support networks immensely. The Prime Minister was featured in one article asking other New Zealanders to think of mothers needing to give birth at this time (Devlin, 2020), referencing her own birth approximately two years ago and knowing “how it felt.” Some women reported feeling anxious in response to suggestions that they should consider home birth when they had been planning to birth in a hospital, while others who had planned to birth at home were equally as anxious that a midwife might not be available for their planned home birth (Biddle, 2020). The NZCOM reported via the media that they were fielding many inquiries about options for home birth (NZCOM, 2020a) whilst organizations, such as Home Birth Aotearoa, providing support to parents wishing to birth at home were inundated with requests for equipment and advice (Hannagan, 2020).

### Support Persons and Visitors

The experiences of fathers (and in some cases grandmothers) were also reported. For fathers, not being allowed to visit their partner or baby from soon after birth till discharge was extremely stressful. One tragic incident involved the despair one father faced when his partner was transferred to the ICU and he could visit only in the final hours prior to her death, which was presumed to be from a blood infection following cesarean section and stillbirth at 21 weeks gestation (ND, 2020). In contrast, many women reported having an enjoyable time with maternity units quiet and without the normal intensity of visitors. Two articles talked about a baby “lockdown reunion” when Level 4 restrictions were lifted and extended families could reunite, and how novel the experience would be for them to talk about in the future (Steyl, 2020). Some women were disappointed that their babies would have no photographic memories of family around them at birth, although there was good evidence that social media networks were used as a substitutes for the lack of physical contact during this phase (Shaskey, 2020; Wilson, 2020). Apart from one woman claiming that maternity hospital staff “seemed confused” about visiting hours and regulations, when women’s perspectives of midwifery care (both community and core) were discussed, views were positive (Steyl, 2020).

### Midwives’ Experiences

The total number of news articles that focused on midwives’ work during the early stages of the New Zealand Covidian experience were few compared to the focus on clients’ experiences. Sixteen stories of the 71 directly addressed midwifery service. Twelve discussed midwifery workforce issues. Of these, three reported the shortage of PPE for midwives, while most of the remainder discussed how midwives were needing to change their service delivery system during lockdown to phone consultations and reduced in-person appointment times. The telephone appointments were meant to reduce the length of in-person visits when physical distancing was being advised. At times, appointments consisted of blended visits in which most of the appointment could be done by telephone consultation followed by actual physical examination, if required, done within the 15 min guideline to minimize potential exposure to the virus. Apart from the two articles that directly quoted community-based midwives—one was positive, saying “It’s all plain sailing,” whereas the other discussed the midwives’ lack of PPE—the NZCOM was the message source. When quoted, NZCOM was supportive of both midwives and women. For example, NZCOM recommended that women consider home birth, but equally reassured them that choice was theirs, and that wherever they chose to birth, they would be supported. One story that focused on midwives providing exceptional care was in Queenstown, where a local dental clinic had been set up to provide a birth space, as the normal unit was unavailable (Jamieson, 2020). The clinic had a temporary street-side shower added, which was unacceptable, so local midwives negotiated for their clients to be accepted into a hotel to birth in more congenial surroundings. The midwives themselves paid the reduced room fee.

#### Extending Our Search

Although not part of our initial search strategy, several other media outlets did highlight the midwifery situation further; we determined that these outlets needed to be included in this section. Midwives’ desperate pleas for basic equipment such as PPE were evident in these media, adding to the fears of an already fearful public: “Midwives have made pleas on social media for industries no longer needing face masks, gloves and other personal protective gear, to donate or sell them” (Akoorie, 2020, Para 2). This extra work was highlighted as a serious issue during COVID-19: “It’s concerning the extra lengths midwives are having to go to—just to look after their new mums, or mothers-to-be” (Hawkesby, 2020, para 1). There was already public awareness of the long-term struggles of midwives to be recognized for the work they do, and a reporter suggested that the pandemic “has only made it harder to be a midwife” because midwives “constantly having to explain changes to her women, when she’s still trying to get information herself, has been hard” (Writes, 2020, para 21).

Despite midwives reported to have continued to support women in both the hospital and community environments, it was acknowledged that this support came at a cost. The pandemic has exposed a midwifery workforce already under stress and not recognized for the public health services these birth workers provide, and has potentially been “a tipping point for midwives” (Burrows, 2020, para 29). The style and content of reporting of midwifery services in the media during this period reflected the current context in which they are situated in the health system. Their limited visibility as a public health service fostering the wellbeing of families and the wider community resulted in a slow Ministry of Health and District Health Boards engagement with the profession to enable this core service to continue safely.

## Discussion: Birth Choices and Midwifery Practice

The overall impressions of the media responses analyzed herein reveal the shaping of the New Zealand-generated public media discourse around birth during the most acute period of COVID-19 in NZ at the time of initial writing (September 2020). Our media content analysis has provided evidence of what news and commentaries the New Zealand public had available to them via the media that enabled them to construct their own perceptions. The greatest number of relevant articles appeared from February 2020 to April 2020. By May 2020, there were very few such articles appearing in the media sources we analyzed. It is fair to say that while media focus valued the experience and voice of the midwifery client and chose to foreground this in the main, any successes or difficulties midwives themselves were experiencing were communicated only through NZCOM, with few exceptions.

### Birth Choices

The array of choices of birth place—home, primary small community hospitals, standalone birth centers, large hospitals—continued to be supported as safe, accessible and acceptable options, and were actively promoted throughout lockdown by NZCOM, midwifery leaders, the Royal Australia and New Zealand College of Obstetricians and Gynaecologists (RANZCOG) and many LMC midwives, and were reflected in the related media coverage. In addition, in NZ breastfeeding was at no time discouraged and all efforts were made to ensure that immediate mother-baby skin-to-skin contact and breastfeeding were supported, even for mothers who tested COVID-positive (Lowe and Bopp, 2020). Some midwives have reported up to a quarter of their wāhine/women birthing at home during Alert Level 4 (Bathgate, 2020; Biddle, 2020; personal communication from NZCOM Wellington regional meetings). We know that in the pre-COVID context, most New Zealand wāhine gave birth at a secondary facility—a hospital with special care for neonates (40.5%) or a tertiary maternity facility (which includes more extensive neonatal services and additional specialist services for inpatients) (45.5%); 10.5% of wāhine gave birth in a primary facility such as a standalone birth center or small community hospital; and 3.6% of wāhine had home births. These rates have been stable over the last 10 years (MoH, 2018). At the time of this writing, data for the COVID-19 period is not yet available and even anecdotal evidence seems unclear, so no conclusions about trends and differences in rates of out-of-hospital births can be made with confidence.

Initially there were some restrictions on birth partners’, support persons’ and others’ (friends and whānau) attendance at hospital births (Brookes, 2020; Moore, 2020)^3^. There have been anecdotal stories from women of all ethnicities of feeling isolated, lonely and even traumatized by the visitor restrictions, particularly when partners were sent away so quickly after the birth. Some women chose to birth at home as a way of having their partner with them and avoiding breaking their bubble. Likewise, there have been reports of women choosing to go home from hospitals sooner than they would have liked. There was regional variation on how this aspect of postnatal care materialized. For example, one rural standalone birth center north of Auckland reported an increase in postnatal stays of women birthing elsewhere, then immediately transferring to the birth center so partners could be directly involved (personal communication, WWBC directors)^4^. The impacts on women and whānau of having restrictions around the presence of partners and support people during maternity admissions need to be more fully explored to avoid unnecessary harm (For such explorations, see the articles by Thayer and Gildner, Rivera, DeYoung and Mangum, Reyes, Rudrum, and Ozhiganova in this Special Issue).

Having significant others near at birth is a particularly culturally sensitive aspect for Indigenous Māori/Pasifika communities, for whom being with extended family is part of birthing culture. Despite many reports of positive experiences, some women did get separated from partners, whānau and significant others, with yet unknown negative cultural, spiritual and psychosocial consequences—especially for Māori and Pasifika childbearers, who perhaps felt less empowered to speak out or exercise their choices, or lacked the resources to ensure that their needs were addressed in the context of the prevailing Eurocentric medical and political responses to the pandemic.

### Foregrounding Midwifery Practice in a Pandemic

Our study has clearly demonstrated a resilient primary maternity service in which midwives have continued to provide partnership-oriented midwifery care, both in the community and in hospitals in the face of an emerging pandemic. Midwives’ responses were characterized by a commitment to continue providing their services, underpinned by strong interactional and relational components, in both the hospital and community environments, whilst also trying to protect themselves and their clients. In New Zealand and elsewhere, midwives’ efforts, passion, commitment, and skills required to maintain the best possible quality service in a uniquely challenging situation must not be forgotten or minimized in the complex milieu of pandemic health system responses. The extent of the embeddedness of the NZ maternity care system in the community is likely to be a contributing factor to this resilience. Yet it is imperative that added stress to midwives’ workloads be acknowledged, as they were often the only health provider in contact with families throughout lockdown. Moreover, it has been highlighted elsewhere (including in most of the articles in this Special Issue) that the pandemic has hit women harder, partly because essential workers (such as midwives) are mostly female (Lim, 2020). Ensuring ongoing sustainability of maternity care during the pandemic requires the additional workloads of midwives during a pandemic to be resourced appropriately—for example with adequate PPE and payment equity.

Disputes around the value of women’s work and its relative invisibility are historical and ongoing. For example, the “indeterminate” work of French women professionals has suggested that understanding occupational knowledge is a balance between indeterminacy and technicality (Jamous and Peloille, 1970). Jamous and Peloille described “technicality” as explicit knowledge using rules, protocols and taught skills (e.g., how to do an abdominal palpation to determine fetal growth and position), whereas “indeterminate knowledge” refers to tacit and private knowledge that resists rule-based protocols and measurable descriptions (e.g., forming relationships over time to build trust and safety, as midwives do). Comparing the pandemic responses of family physicians and midwives illustrates this point. The New Zealand government provided immediate funding of NZ $30 million ($19 million USD) for family physicians and pharmacists on the 2^nd^ of April, yet the cost of the increased workload for midwives was not acknowledged until after the initial lockdown. Although family physicians of course are both male and female, the general perception of the medical profession in NZ is one of male domination, inferring ways of knowing and skills that are quite different from those of midwives, who are almost all women. Jamous and Peloille’s seminal work reveals how women’s work can be caught in a relentless, self-perpetuating system that fails to acknowledge women’s professionalism and sees their work as less important than men’s.

Arguably, much has changed for women and midwives over the last 50 years, yet many of these issues continue. Kirkham (2015) study of midwives in the UK showed that midwifery is often at best misunderstood and at worst exploited, leading to adversarial conditions for the professional midwife. Kirkham argued that midwifery work is often not prioritized because relationships and care are not counted as measurable commodities and therefore get afforded less value and are overlooked by economic and political systems. This focus undervalues midwives’ significant emotional work of building and maintaining relationships. Yet it is well-established that relationships built and sustained over time enable intuitional ways of knowing that facilitate trust and safety (Davis-Floyd and Elizabeth, 1996; Crowther and Smythe, 2016). Furthermore, it is these relationships with women and families that support and sustain midwives’ professional autonomy and their resultant enjoyment of practice (Clemons et al., 2020).

In the context of the pandemic, midwives are caught in a competitive fiscal environment that does not prioritize the significance of relationships, despite their obvious centrality to women’s experiences as described in the media. Accepting that the core NZ midwifery value is *partnership*—the embeddedness of relationships with women, families and communities—suggests that any attempt to quantify in monetary terms the significance and value of this relational work could be detrimental (Davies et al., 2019). Yet it is vital that this relational work be afforded monetary value for midwives to be paid appropriately. The COVID-19 lockdown highlighted how midwives, women and communities are invested in these relationships despite the fiscal constraints caused by insufficient governmental investment in midwifery. The NZ midwife is proving to be a major influencer and initiator for relational care to occur uninterrupted at the frontline throughout the COVID-19 lockdown, despite the personal risk. Midwives kept women, their families and communities central to the conversation throughout lockdown, whilst juggling concerns about keeping themselves and their own families safe.

Women were at no point required to be COVID tested before birth. Everyone accessing healthcare is routinely asked screening questions, and staff apply PPE or test accordingly. This may relate to the fact that community transmission of the virus has been well-controlled in NZ. New Zealand midwives were not required to wear full PPE at home and in birth centers unless the wāhine or their whānau were either positive for COVID or been exposed and were awaiting test results. In these situations, women were advised to birth in the hospital. The recommendation for home and birth center settings was to provide care as normal using PPE as appropriate and to maintain physical distancing from other members of the whānau and support persons as much as is feasible in these primary settings. Each midwife determined the risk and donned PPE accordingly as the professional guidance evolved over time. Yet in the early phase of the pandemic, the ambiguous and changing guidance and lack of available PPE caused anxiety for many midwives.

With multiple job losses related to the pandemic for many workers, midwifery was able to establish itself more visibly as a core service and a secure career prospect. This positive Covidian outcome is offset by stress, low pay and midwives departing the workforce for these reasons—a factor that has a significant impact on recruitment to undergraduate midwifery programs. However, there could be a shift in that image, as a momentous boost to the maternity system was announced as we were finalizing this article: NZ maternity services will receive an extra NZ $180 million ($118 million USD), including a pay raise for midwives and particular consideration towards rural midwifery and the rising complexities related to birth. This is very good news for midwives and for the entire NZ maternity care system.

It is essential that NZ women, families and communities continue to receive quality equitable care across all regions. Therefore, attracting new midwives to the profession will be necessary to enable the workforce to strengthen and replace those for whom “enough was enough.” Although COVID-19 brought heightened vigilance for personal safety and served to magnify midwives’ employment conditions and pay inequities, there was never any doubt that women would be left without their midwives or be coerced into childbirth choices they did not want.

Despite the sudden challenges imposed by COVID-related lockdowns, midwives across NZ provided high quality, individualized care with passion and commitment, and continued to facilitate choice to the best of their capability within the imposed restrictions and resource concerns—which at times may have come at personal and professional costs. Ongoing informal communications about clinical practice concerns and how to ensure continuity of care whilst keeping the whānau/families they serve, themselves and their own whānau safe have been continuously highlighted in the NZ media. What was apparent when examining multiple media sources was lack of preparedness and an avalanche of changing guidance, leaving midwives vulnerable. This was set against a backdrop of a workforce that often already felt marginalized and unappreciated by policy makers and politicians, in which idiosyncratic midwifery relational knowing continues to be subjugated by dominant gender, neoliberal and biomedical discourses.

## Strengths and Limitations

The strength of our study is that it has been conducted close to the phenomena being explored both in context and time. At the initial time of writing (September 2020), a further regional lockdown was occurring, highlighting the significance of this genre of work to ensure that voices and learnings are not lost. We note again the limitation of excluding *The Herald* database in our counting of articles for the media content analysis. We do believe that by doing this we have avoided any potential error confounded by duplication. Another limitation is our application of Western research methods, theoretical concepts, knowing and paradigms. These do not privilege *Te Tiriti o Waitangi* nor Indigenous knowledge; as New Zealand researchers we must acknowledge this as a limitation to our exploration. Media content analysis is a valuable and insightful telling of various stories, but the degree to which this also reflects the true lived experiences of midwifery practice, or indeed the cultural diversity of NZ, cannot be validated and requires further examination.

## Conclusion: The Need for Full Recognition of Midwives’ Contributions

Whilst the world continues to grapple with the COVID-19 pandemic, NZ is now in the fortunate position of low infections and deaths relative to many other high-income countries. At the final time of writing (January 2021), we have continued to experience regional lockdowns, so conclusions about changes to outcomes or/and variation of choices of care can only be tentative. The premise of our article was to illuminate the complexity and efforts of New Zealand midwives during lockdown; however, based on the discussion points raised in combination with the content analysis of contemporaneous experiences reported in the media, an array of future related research is warranted. It is clear that midwives must be fully recognized for their contributions to society and for how their work influences the very warp and weave of NZ’s social fabric—especially in a national crisis. Our media content analysis has contributed to foregrounding midwives’ contributions. As Davies et al. (2019: 245) conclude:

[The] continuity of care model works on the premise that the underlying philosophy of the midwifery profession supports a community based primary health service that strengthens family relationships and promotes normal birth…[this] supports the principles of social sustainability such as equity, social justice, community capacity…[and meets] the cultural and spiritual needs of women, their babies and families.

It is this social connectivity that enables women and their families to extend beyond prior expectations of childbirth and to flourish even in profoundly disruptive times of crisis. The COVID-19 disruptions further reveal what midwives in NZ aspire to do—which is to ignite the power that already and always rests with women through relationship-focused care. To be clear, women and midwives need not be empowered—they already and always have been empowered; they just need the circumstances for their power to emerge and flourish. Midwives have continued to provide their clients with exemplary and creative solutions to the unrelenting pandemic-imposed challenges throughout COVID-19 in NZ.

Irrespective of the global fear-based rhetoric and misinformation concerning maternity care, it is clear that NZ midwives, like the Puerto Rican, US, Canadian, Mexican, Chilean, Kenyan, Pakistani, and Guatemalan midwives described in other articles in this issue, showed fortitude during the worst of COVID-19 in NZ. Our media content analysis has revealed how NZ midwives have continued to the best of their ability in times of adversity to provide evidence based relational care that places women, their families and communities at the center of all care decisions. Long-term insights from this media analysis suggest that relational continuity facilitates quality and consistent care that honors women’s choices, cultural needs, and human rights even during situations of national crisis. The healthcare needs of women, especially childbearing women, must remain priorities during pandemics and other disasters. The dedication of frontline midwives deserves our praise and appreciation. Midwives need to be heard, seen, understood, and treasured by policy makers and politicians. Only then can our midwifery colleagues receive the strong governmental mandate needed to continue to undertake their powerful and valuable work for society, including in times of crisis.

## Data Availability

The datasets presented in this article are not readily available because Media sources available on linked reference list. Requests to access the datasets should be directed to susan.crowther@aut.ac.nz

## Footnotes

1 Two key concepts characterized the New Zealand COVID-19 response: the national Four-Level Alert System and the “bubble” concept. Initially developed by a group of medical researchers advising New Zealand’s Ministry of Health on how to protect those with disabilities from COVID-19, the bubble concept became a mainstay of communication around how New Zealanders could minimize coronavirus transmission. A person’s “bubble” is essentially their household; at times of increased risk of virus transmission, New Zealanders are encouraged to “stay in your bubble” or “don't break your bubble.”

2 In order to retrieve articles from the Stuff website, we posted a combination of search terms into the Stuff website search option. These included “COVID + Birth” “COVID + Pregnancy”, “Corona + Birth”, “Corona + pregnancy” and “midwifery/ midwives + COVID.”

3 Doulas are not part of New Zealand maternity culture, partly due to the emphasise on midwives providing continuity of carer across jurisdictions and providing the relational continuity that doulas have been shown to provide in other regions.

4 This refers to the Warkworth Birth Center, which has a unique setup, and thus may give the impression that all women can have this type of postpartum of care in New Zealand, which is unfortunately untrue. The NZ government does not provide resources for all families to experience this level of postnatal care provision. We mention it here to illustrate the extent of regional differences apparent across New Zealand.

## References

[B1] Akoorie N. (2020). Midwives have made please on social media for industries no longer needing face masks, gloves and other personal protective gear, to donate or sell them. NZ Herald (Accessed March 25, 2020).

[B2] Bathgate B. (2020). Coronavirus: new life, and new birthing procedures, amidst the lockdown. Stuff Available at: https://www.stuff.co.nz/national/health/coronavirus/120792144/coronavirus-new-life-and-new-birthing-procedures-amidst-the-lockdown (Accessed April 5, 2020).

[B3] Berinstein J. Ellen B. Guilliand K. (2020). Valuing the labour of midwives in Ontario, Canada and New Zealand. Sustainability, Midwifery and Birth 64–80. 10.4324/9780429290558-4

[B4] Biddle D.-L. (2020). Coronavirus: lack of protective gear means women may be forced to birth alone. Stuff (Accessed March 31, 2020).

[B5] Brookes E. (2020). Lockdown birth: no visitors, epidural in doubt. Stuff (Accessed March 28, 2020).

[B6] Burrows M. (2020). Coronavirus: midwives leaving their jobs 'in droves' as COVID-19 exposes culture of overwork, stress, poor pay. Newshub (Accessed May 13, 2020).

[B7] Clemons J. H. Gilkison A. Mharapara T. L. Dixon L. McAra-Couper J. (2020). Midwifery job autonomy in New Zealand: i do it all the time. Women and Birth 34, 30–37. 10.1016/j.wombi.2020.09.004 32962945

[B8] Crowther S. Smythe L. (2016). Open, trusting relationships underpin safety in rural maternity a hermeneutic phenomenology study. BMC Pregnancy Childbirth 16 (370), 370. 10.1186/s12884-016-1164-9 Available at: http://rdcu.be/m0cN. 27881105 PMC5122205

[B9] Davies L. Crowther S. Hunter B. (2019). “Midwifery continuity of care: theorising towards sustainability,” in Midwifery continuity of care. Editors Homer C. Brodie P. Sandall J. (Melbourne, Australia: Elsevier), 231–248.

[B10] Davies L. Crowther S. (2020). The midwife as social connector. Sustainability, Midwifery and Birth, 83–98. 10.4324/9780429290558-5

[B11] Davis-Floyd R. Elizabeth D. (1996). Intuition as authoritative knowledge in midwifery and home birth. Med. Anthropol. Q. 10 (2), 237–269. 10.1525/maq.1996.10.2.02a00080 8744086

[B12] Devlin C. (2020). Coronavirus: Prime Minister Jacinda Ardern urges people be mindful of new mums. Stuff (Accessed March 29, 2020).

[B13] Farry A. Crowther S. (2014). Cultural safety in New Zealand midwifery practice. Part 2. Pract. Midwife 17 (7), 30–33. 25109074

[B14] Flenady V. Aleena M. Middleton P. Ellwood D. Erwich J. J. Coory M. et al. (2016). Stillbirths: recall to action in high-income countries. The Lancet 387 (10019), 691–702. 10.1016/S0140-6736(15)01020-X 26794070

[B15] Gilkison A. Pairman S. McAra-Couper J. Kensington M. James L. (2016). Midwifery education in New Zealand: education, practice and autonomy. Midwifery 33, 31–33. 10.1016/j.midw.2015.12.001 26719195

[B16] Guilliland K. Pairman S. (2010). The midwifery partnership. 2nd Edn. Christchurch, New Zealand: New Zealand College of Midwives.

[B17] Hannagan S. (2020). What Covid-19 means for women giving birth in Aotearoa: home birth aotearoa. Available at: https://homebirth.org.nz/what-covid-19-means-for-women-giving-birth-in-aotearoa/ (Accessed March 30, 2020).

[B18] Hawkesby K. (2020). Midwives in crisis during Covid 19 coronovirus pandemic. NZ Herald (Accessed April 6, 2020).

[B19] HDSR (2020). Final report – Pūrongo Whakamutunga: health and disability system review. Available at: https://systemreview.health.govt.nz/assets/Uploads/hdsr/health-disability-system-review-final-report.pdf (Wellington).

[B20] Herald N. Z (2020). Covid 19 coronavirus: cash injection brings relief but anxiety over long-term funding remains. NZ Herald (Accessed June 30, 2020).

[B21] Hunter M. Crowther S. McAra-Couper J. Gilkison A. MacGregor D. Gunn J. (2016). Generosity of spirit sustains caseloading Lead Maternity Carer midwives in New Zealand. New Zealand College of Midwives 52, 50–55. 10.12784/nzcomjnl52.2016.8.50-55

[B22] Jamieson D. (2020). Coronavirus: Queenstown lockdown baby born in a four-star hotel. Stuff (Accessed April 6, 2020).

[B23] Jamous H. Peloille B. (1970). “Professions or selfperpetuating systems?’ Changes in the French university-hospital system,” in Professions and Professionalisation. Editor Jackson J. A. (Cambridge, UK: Cambridge University Press).

[B24] Jefferies S. French N. Gilkison C. Graham G. Hope V. Marshall J. (2020). COVID-19 in New Zealand and the impact of the national response: a descriptive epidemiological study. Lancet Public Health 5 (11), e612–e623. 10.1016/S2468-2667(20)30225-5 33065023 PMC7553903

[B25] Kirkham M. (2015). Commodification around birth: sociology for midwives. Cambridge, UK: Polity Press.

[B26] Lim E. (2020). COVID-19 BRIEF: impact on women and girls. US Global Leadership Coalition (Accessed August 10, 2020).

[B27] Lowe B. Bopp B. (2020). COVID‐19 vaginal delivery–a case report. Aust. N. Z. J. Obstet. Gynaecol. 60, 465–466. 10.1111/ajo.13173 32294229 PMC7262173

[B28] MCNZ (2019). Annual report of the midwifery Council of New Zealand. Wellington, NZ: Midwifery Council Te Tatau o te Whare Kahu Available at: https://www.midwiferycouncil.health.nz/common/Uploaded%20files/Annual%20reports/Midwifery%20Council%20Annual%20Report%202019.pdf.(Wellington).

[B29] MCNZ (n.d.). Concerns about a midwife Wellington. Wellington, NZ: Midwifery Council of New Zealand Available at: https://www.midwiferycouncil.health.nz/ (Accessed December 2020).

[B30] Midwfery Council (2019). 2019 Midwifery workforce survey. Wellington, NZ: MCNZ

[B31] MoH (2020a). COVID-19 (novel coronavirus). Available at: https://www.health.govt.nz/our-work/diseases-and-conditions/covid-19-novel-coronavirus (Accessed August 8, 2020). Ministry of Health.

[B32] MoH (2020b). Fetal and infant deaths web tool. Wellington, NZ: Ministry of Health.

[B33] MoH (2011). New Zealand Maternity Standards: a set of standards to guide the planning, funding and monitoring of maternity services by the Ministry of Health and District Health Boards. Wellington, NZ: Ministry of Health.

[B50] MOH (2018). Report on maternity (2016). Wellington: Ministry of Health.

[B34] MoH (2019). Report on maternity 2017. Wellington, NZ: Ministry of Health.

[B35] Moore R. (2020). Coronavirus: a Manawatu dad is separated from his premature baby in Wellington Hospital. Stuff (Accessed April 5, 2020).

[B36] Myllylahti M. Baker S. (2019). JMAD (Journalism, Media and Democracy) New Zealand Media Ownership Report 2019. Auckland: AUT.

[B37] ND. (2020). Auckland DHB investigating after increase in maternal deaths. Stuff (Accessed May 30, 2020).

[B38] NZCOM (2020a). Media release: midwives baffled and disappointed by lack of govt financial support. Aotearoa, NZ: New Zealand College of midwives Available at: https://www.midwife.org.nz/news/midwives-baffled-and-disappointed-by-lack-of-govt-financial-support/ (Accessed April 21, 2020).

[B39] NZCOM (2020b). Special Covid-19 online edition. Aotearoa, NZ: Midwife Aotearoa New Zealand. (Accessed June, 2020).

[B40] NZIER (2020). Sustainable midwifery: supporting improved wellbeing and greater equity. Christchurch, New Zealand: NZCOM.

[B41] O’Connell M. Crowther S. Ravaldi C. Homer C. (2020). Midwives in a pandemic: a call for solidarity and compassion. Women and birth 33 (3), 205–206. 10.1016/j.wombi.2020.03.008 32241720 PMC7270587

[B42] Pairman S. McAra-Couper J. (2015). “Theoretical framewworks for midwifery practice,” in Midwifery: preparation for practice. Editors Pairman S. Pincombe J. Thorogood C. Tracy S. K., (Elsevier Health Sciences APAC), 386–411.

[B52] PMMRC (2018). Twelfth annual report of the perinatal and maternal mortality review committee: reporting mortality 2016. Wellington: Health Quality & Safety Commission.

[B43] Robert A. (2020). Lessons from New Zealand's COVID-19 outbreak response. Lancet Public Health 5 (11), e569–e570. 10.1016/S2468-2667(20)30237-1 33065024 PMC7553856

[B44] Rowland T. McLeod D. Froese-Burns N. (2012). Comparative study of maternity systems. Wellington, NZ: Malatest International consultant and advisory services.

[B45] Shaskey T.(2020). Coronavirus: 'Scary and uncertain time' for mothers and midwives. Stuff Available at: https://www.stuff.co.nz/taranaki-daily-news/news/120847610/coronavirus-scary-and-uncertain-time-for-mothers-and-midwives (Accessed April 13, 2020).

[B46] Stats N. Z (2020). Stats NZ Tatauranga Aotearoa. Population. Available at: https://www.stats.govt.nz/topics/population (Accessed August 5, 2020).

[B47] Steyl L. (2020). Fond memories for parents of lockdown babies. Stuff Available at: https://www.stuff.co.nz/life-style/121933230/fond-memories-for-parents-of-lockdown-babies. (Accessed June 25, 2020).

[B51] Vermeer S. Trilling D. Kruikemeier S. de Vreese C. (2020). Online news user journeys: the role of social media, news websites, and topics. Digital Journalism 8 (9), 1114–1141.

[B48] Wilson L. (2020). Baby Anahera made her entrance just before lockdown, providing 'good news in a stink time. Stuff Available at: https://www.stuff.co.nz/life-style/parenting/baby/120651826/baby-anahera-made-her-entrance-just-before-lockdown-providing-good-news-in-a-stink-time.

[B49] Writes E. (2020). Covid-19 has only made it harder to be a midwife. Spinoff (Accessed May 5, 2020).

